# Probing the Interactions of Ochratoxin B, Ochratoxin C, Patulin, Deoxynivalenol, and T-2 Toxin with Human Serum Albumin

**DOI:** 10.3390/toxins12060392

**Published:** 2020-06-13

**Authors:** Zelma Faisal, Virág Vörös, Eszter Fliszár-Nyúl, Beáta Lemli, Sándor Kunsági-Máté, Rita Csepregi, Tamás Kőszegi, Ferenc Zsila, Miklós Poór

**Affiliations:** 1 Department of Pharmacology, Faculty of Pharmacy, University of Pécs, Szigeti út 12, H-7624 Pécs, Hungary; faisal.zelma@gytk.pte.hu (Z.F.); viru@gmail.hu (V.V.); eszter.nyul@aok.pte.hu (E.F.-N.); 2 János Szentágothai Research Centre, Ifjúság útja 20, H-7624 Pécs, Hungary; beata.lemli@aok.pte.hu (B.L.); sandor.kunsagi-mate@aok.pte.hu (S.K.-M.); ritacsepregi93@gmail.com (R.C.); koszegi.tamas@pte.hu (T.K.); 3 Institute of Organic and Medicinal Chemistry, Medical School, University of Pécs, Szigeti út 12, H-7624 Pécs, Hungary; 4 Department of Laboratory Medicine, University of Pécs, Medical School, Ifjúság útja 13, H-7624 Pécs, Hungary; 5 Institute of Materials and Environmental Chemistry, Research Centre for Natural Sciences, Magyar Tudósok krt. 2, H-1117 Budapest, Hungary; zsila.ferenc@ttk.mta.hu

**Keywords:** ochratoxin B, ochratoxin C, patulin, deoxynivalenol, T-2 toxin, human serum albumin, fluorescence spectroscopy, optical spectroscopy, albumin–ligand interaction, cytotoxicity

## Abstract

Ochratoxins, patulin, deoxynivalenol, and T-2 toxin are mycotoxins, and common contaminants in food and drinks. Human serum albumin (HSA) forms complexes with certain mycotoxins. Since HSA can affect the toxicokinetics of bound ligand molecules, the potential interactions of ochratoxin B (OTB), ochratoxin C (OTC), patulin, deoxynivalenol, and T-2 toxin with HSA were examined, employing spectroscopic (fluorescence, UV, and circular dichroism) and ultrafiltration techniques. Furthermore, the influence of albumin on the cytotoxicity of these xenobiotics was also evaluated in cell experiments. Fluorescence studies showed the formation of highly stable OTB–HSA and OTC–HSA complexes. Furthermore, fluorescence quenching and circular dichroism measurements suggest weak or no interaction of patulin, deoxynivalenol, and T-2 toxin with HSA. In ultrafiltration studies, OTB and OTC strongly displaced the Sudlow’s site I ligand warfarin, while other mycotoxins tested did not affect either the albumin binding of warfarin or naproxen. The presence of HSA significantly decreased or even abolished the OTB- and OTC-induced cytotoxicity in cell experiments; however, the toxic impacts of patulin, deoxynivalenol, and T-2 toxin were not affected by HSA. In summary, the complex formation of OTB and OTC with albumin is relevant, whereas the interactions of patulin, deoxynivalenol, and T-2 toxin with HSA may have low toxicological importance.

## 1. Introduction

**Figure 1:**
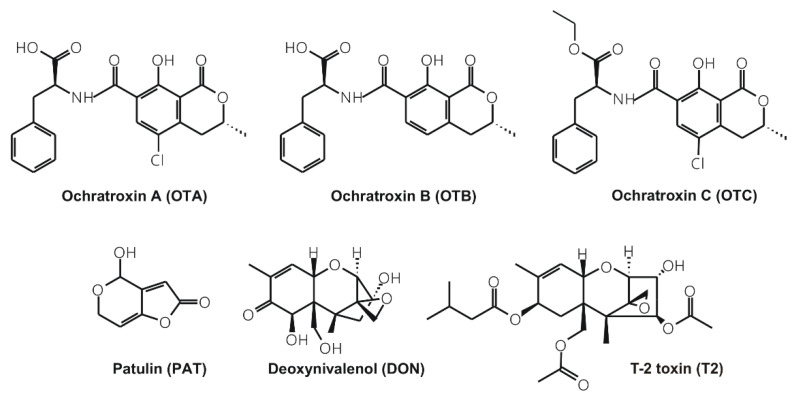
Chemical structures of ochratoxin A (OTA), ochratoxin B (OTB), ochratoxin C (OTC), patulin (PAT), deoxynivalenol (DON), and T-2 toxin (T2).

Mycotoxins are secondary fungal metabolites, which commonly contaminate food and feed, causing different toxic effects in animals and humans [1]. Ochratoxins are produced by *Aspergillus* and *Penicillium* species, containing a dihydroisocoumarin moiety linked to L-phenylalanine (Figure 1) [2]. These nephrotoxic agents appear as contaminants in grains, cereal-based commodities, fruits, spices, and beverages (e.g., wine, beer, milk, and coffee) [3,4]. Among ochratoxins, ochratoxin A occurs most frequently; however, its dechlorinated and ethyl ester derivatives—ochratoxin B (OTB) and ochratoxin C (OTC), respectively—seem to be also important (Figure 1). OTC is a similarly toxic to ochratoxin A, while OTB is less toxic [4]. Patulin (PAT; Figure 1) is produced by *Aspergillus* and *Penicillium* species. Due to its antiviral, antifungal, and antibacterial activities, PAT was used in the treatment of common cold and skin infections in the 1940s. However, it has been classified as a toxin from the 1960s, due to its harmful effects [5]. PAT frequently appears in blue mold contaminated apples, pears, and related products (e.g., apple juice, compotes, ciders, and baby food puree) [6]. Excitement, convulsions, dyspnea, pulmonary congestion, edema, ulceration, and gastrointestinal tract distension can be the symptoms of acute PAT intoxication [7]. After chronic exposure, PAT may induce genotoxic, teratogenic, and immunotoxic effects [6]. Trichothecene mycotoxins (containing 2,13-epoxytrichothene moiety) are produced by several filamentous fungi, including *Fusarium, Myrothecium, Phomopsis, Stachybotrys*, and *Trichoderma* species [5]. Deoxynivalenol (DON or also called as vomitoxin; Figure 1) is one of the most common trichothecene contaminants in cereals (e.g., wheat, barley, corn, and rye) and in related products (e.g., flour, bread, and beer) [5]. Low dose intake of DON can lead to weight loss, whereas high doses exert gastrointestinal disorders (e.g., nausea, vomiting, and diarrhea) [5,8]. Chronic exposure to DON induces cytotoxic, immunotoxic, and genotoxic effects as well as reproductive disorders [9]. Among trichothecenes, T-2 toxin (T2; Figure 1) is one of the most cytotoxic [10]. T2 can provoke immunosuppressive, gastrointestinal, dermatological, and neurological symptoms [5,11,12,13].

Human serum albumin (HSA) is the dominant circulating plasma protein. Albumin–ligand interactions result in the partial entrapment of ligand molecules, which may influence the toxicokinetics of HSA-bound xenobiotics [14,15]. Most of the compounds occupy one of the three major drug binding sites on HSA: Sudlow’s site I, Sudlow’s site II, and the heme binding site (or FA1) [14,16]. Albumin binds tightly several mycotoxins, including alternariol, citrinin, ochratoxin A, and zearalenone [17,18,19,20]. The interaction of ochratoxin A with HSA has been extensively studied [17,21,22,23], however, only limited data are available regarding other ochratoxins. Despite the fact that these mycotoxins frequently appear in food and animal feed (and consequently, in the body fluids of humans and animals), the interactions of OTB, OTC, DON, PAT, and T2 with HSA were not or only barely investigated. Previous reports suggest that OTB, DON, and PAT bind to the site I (subdomain IIA) of HSA, showing approximately 1.8 × 10^6^ L/mol [22], 4.6 × 10^4^ L/mol [24], and 1.4 × 10^4^ L/mol [25] association constants, respectively. Only one individual study was reported regarding each mycotoxin–HSA interaction, employing spectroscopic and molecular docking studies [22,24,25]. To the best of our knowledge, the interaction of OTC and T2 with HSA has not yet been evaluated. Additionally, some toxicokinetic aspects of mycotoxins are poorly examined and/or understood. In our view, the importance of mycotoxin–albumin complexes is underestimated. Besides the interactions of mycotoxins with biotransformation enzymes and drug transporters, their albumin binding seems to be a relevant factor as well. Considering the stability of the formed complexes, their toxicological importance can be estimated [15,21]; however, other factors should also be considered. Furthermore, the binding location of a mycotoxin on albumin predicts which compounds are able to displace it from the protein. If the albumin-bound fraction of a mycotoxin is high in the circulation, the displacement may cause significant changes in its tissue distribution and/or elimination, and consequently, in its toxicity. In addition, the species differences in albumin binding may help to understand the high species-dependent alternations in the toxicity of certain mycotoxins (e.g., zearalenone and its metabolites) [19,26]. Generally, mycotoxin–albumin interactions are barely examined, and most studies apply only spectroscopic techniques. Despite the fact that fluorescence and UV–Vis spectroscopy are powerful techniques to examine protein–ligand interactions, we need to consider their limitations, which can explain some controversial results reported for some mycotoxins (e.g., aflatoxin B1) [27,28,29]. Therefore, other methodologies are also required to obtain well-established conclusions, and extensive in vitro experiments as well as animal studies need to be performed for deeper understanding of mycotoxin–albumin interactions.

In this study, the complex formation of OTB, OTC, PAT, DON, and T2 with HSA was examined employing fluorescence spectroscopy, ultrafiltration, and cell experiments. Because PAT, DON, and T2 have no intrinsic fluorescence, their interactions were investigated by using circular dichroism (CD) and UV spectroscopy. In previous studies [22,24,25], the complex formation of OTB, PAT, and DON with HSA were examined with spectroscopic techniques. Because the application of other methods is also reasonable to confirm these results, in the current study. Cell experiments are important to test the effect of albumin binding on the cellular uptake and cytotoxicity of xenobiotics. Besides the interaction with albumin, several other factors can influence the tissue uptake of mycotoxins, such as the diffusibility of the compound and the involvement of active transport mechanisms. Thus, even if the binding constant of an albumin–ligand complex is known, cell experiments are useful for the more precise prediction of the importance of albumin binding in tissue uptake. Since hepatocytes express several drug transporters which can be involved in the cellular uptake of mycotoxins, HepG2 liver cells were used in these experiments. Therefore, to test the toxicological importance of the albumin binding of OTB, OTC, PAT, DON, and T2, the effects of fetal bovine serum (10%) and HSA (40 g/L) on the acute in vitro toxicity of mycotoxins were studied.

## 2. Results and Discussion

### 2.1. Fluorescence Spectroscopic Investigation of the Interactions of OTB and OTC with HSA

**Figure 2:**
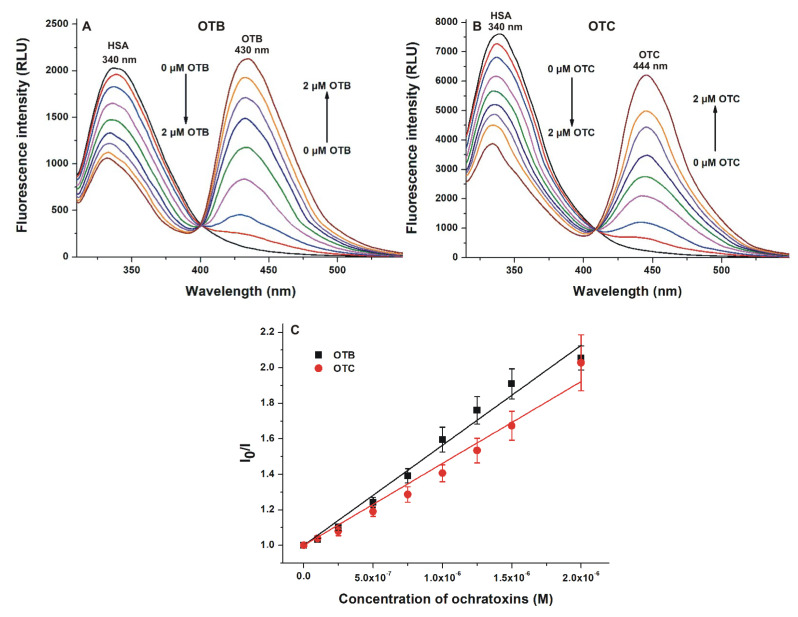
Fluorescence quenching effects of ochratoxins on human serum albumin (HSA). Emission spectra of HSA (2 μM) in the presence of increasing concentrations of ochratoxin B (OTB; (**A**); ex slit = 5 nm, em slit = 10 nm) and ochratoxin C (OTC; (**B**); ex slit = 10 nm, em slit = 10 nm) in phosphate-buffered saline (PBS, pH 7.4; λ_ex_ = 295 nm). Stern–Volmer plots of OTB–HSA and OTC–HSA complexes (**C**) (λ_em_ = 340 nm; RLU: relative light unit).

The fluorescence quenching effects of OTB and OTC on HSA (2 µM) were studied in the presence of increasing mycotoxin concentrations (0–2 µM). OTB and OTC decreased the emission signal of HSA at 340 nm in a concentration-dependent fashion (Figure 2A,B). Since the inner filter effect of ochratoxins was negligible (the corrections were performed based on Equation (1)), these observations suggest the formation of mycotoxin–HSA complexes. Based on the strong quenching effects of OTB and OTC on HSA, these mycotoxins occupy a binding site close to the Trp-214 residue. Therefore, it is reasonable to conclude that the high-affinity binding site of OTB and OTC is located at Sudlow’s site I (subdomain IIA), similar to the parent mycotoxin ochratoxin A [30,31]. The appearance of increasing second peaks at 430 (Figure 2A) and 444 nm (Figure 2B) are due to the intrinsic fluorescence of OTB and OTC, respectively. Stern–Volmer plots of OTB–HSA and OTC–HSA interactions are shown in Figure 2C.

**Figure 3:**
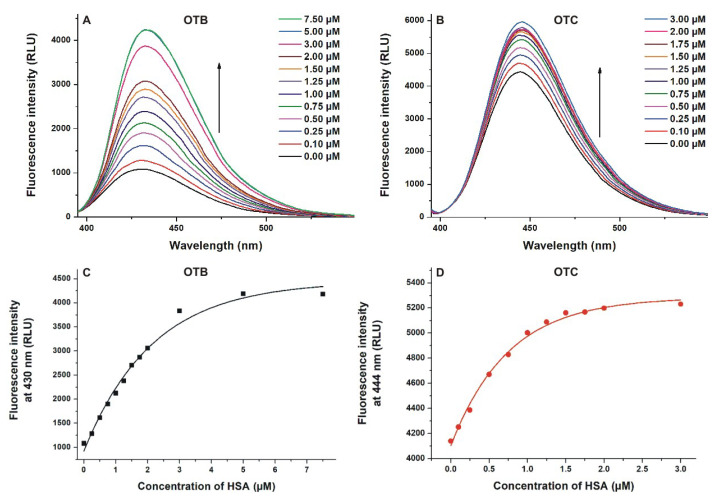
Effects of HSA on the fluorescence of ochratoxins. Fluorescence emission spectra of ochratoxin B (OTB; (**A**); 1 μM; λ_ex_ = 365 nm; ex slit = 5 nm, em slit = 5 nm) and ochratoxin C (OTC; (**B**); 1 μM; λ_ex_ = 380 nm; ex slit = 5 nm, em slit = 10 nm) in the presence of increasing human serum albumin (HSA) concentrations in phosphate-buffered saline (PBS, pH 7.4). HSA-induced increase in the emission intensities of OTB (**C**) and OTC (**D**) at 430 and 444 nm, respectively (RLU: relative light unit). The emission intensity of OTB and OTC did not increase further above 7.5 and 3 μM HSA concentrations, respectively.

**Figure 4:**
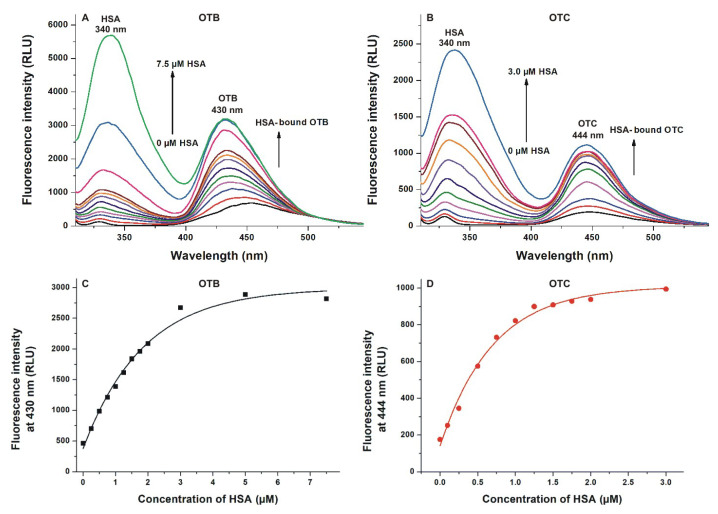
Assessment of ochratoxin–albumin interactions based on energy transfer between Trp-214 and mycotoxins (unlike Figure 3, samples were excited at 295 nm). Fluorescence emission spectra of ochratoxin B (OTB; (**A**); 1 μM; ex slit = 5 nm, em slit = 10 nm) and ochratoxin C (OTC; (**B**); 1 μM; ex slit = 5 nm, em slit = 10 nm) in the presence of increasing human serum albumin (HSA) concentrations in phosphate-buffered saline (PBS, pH 7.4; λ_ex_ = 295 nm). Emission intensities of OTB (**C**) and OTC (**D**) at 430 and 444 nm, respectively: these data were corrected by subtracting the emission signal of HSA at the wavelengths used (RLU: relative light unit). The emission intensity of OTB and OTC did not increase further above 7.5 and 3 μM HSA concentrations, respectively.

**Figure 5:**
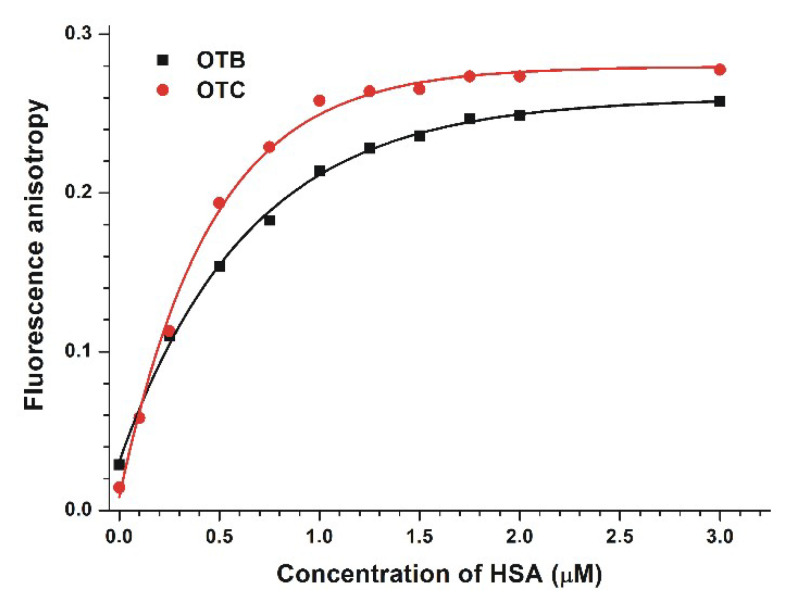
Fluorescence anisotropy values of ochratoxin B (OTB; 1 µM; λ_ex_ = 365 nm, λ_em_ = 430 nm) and ochratoxin C (OTC; 1 µM; λ_ex_ = 380 nm, λ_em_ = 444 nm) in the presence of increasing human serum albumin (HSA) concentrations in phosphate-buffered saline (PBS, pH 7.4; ex slit = 10 nm, em slit = 10 nm). The fluorescence anisotropy of OTB and OTC did not increase further above 3 μM HSA concentration.

In the next step, increasing amounts of HSA (0–7.5 μM) were added to 1 µM mycotoxin. Similarly to the previous report with ochratoxin A and 2′*R*-ochratoxin A [23], these samples were applied for three different fluorescence measurements (marked below as I, II, and III).

(I) First, 365 (OTB) and 380 nm (OTC) excitation wavelengths were used to test the effect of HSA on the intrinsic fluorescence of ochratoxins. Emission signals of both OTB and OTC significantly increased with the elevation of HSA concentration (Figure 3), and the relative increase in the fluorescence of OTB was larger compared to OTC. Importantly, under the applied circumstances, HSA did not show emission signal. These observations suggest again the formation of ochratoxin–albumin complexes. Because the fluorescence of aromatic fluorophores can be partly quenched by water molecules [32], the binding of OTB and OTC to HSA results in the partial decomposition of their hydration shell. Consequently, the quenching effect of water molecules decreases and the emission signal of the mycotoxins rises [18,20,23]. In the presence of HSA, a slight red shift (430 → 435 nm) was observed in the emission wavelength maximum of OTB, as was also reported by Perry et al. [22]. HSA caused a similar shift in the emission spectrum of OTC (444 → 447 nm). As it is demonstrated in Figure 3C,D, lower HSA concentrations were enough to achieve the complete albumin binding of OTC vs. OTB (approximately 2–3 vs. 5–7 μM, respectively), suggesting that HSA forms more stable complexes with OTC.

(II) In the next experiment, fluorescence emission signals of OTB and OTC were examined again; however, the emission spectra were recorded using the excitation maximum of Trp-214 in HSA (295 nm). The first peak, with the maximum at 340 nm, is the emission signal of HSA. Because the fluorescence emission spectrum of Trp-214 overlaps with the excitation spectra of OTB and OTC, and the binding sites of ochratoxins are close to the Trp-214 residue, the energy transfer between HSA and ochratoxins leads to the increase in the emission signal around 430–450 nm (Figure 4A,B). These results also demonstrate the stronger interaction of HSA with OTC compared to OTB (Figure 4C,D).

(III) Finally, fluorescence anisotropy values of the samples were also determined (OTB: λ_ex_ = 365 nm, λ_em_ = 430 nm; OTC: λ_ex_ = 380 nm, λ_em_ = 444 nm). Fluorescence anisotropy is affected by the rotational freedom of the molecules in an inversely proportional manner [33]. The small molecules (e.g., OTB and OTC) possess high rotational freedom accompanied with low anisotropy values. However, the complex formation with HSA decreases their rotational freedom, which leads to a large elevation in anisotropy [23,34]. Fluorescence anisotropy values of OTB and OTC strongly increased in the presence of HSA (Figure 5), confirming their protein binding as well as the higher affinity of OTC.

Based on fluorescence measurements, Stern–Volmer quenching constants (*K_SV_*; unit: L/mol) and association constants (*K_a_*; unit: L/mol) of ochratoxin–HSA complexes were determined (see details in Section 4.2). Both OTB and OTC form highly stable complexes with HSA. Only quenching studies proposed somewhat lower association constants; however, the other three methods showed good correlations, suggesting approximately 10^6^ and 10^7^ L/mol association constants of OTB–HSA and OTC–HSA complexes, respectively (Table 1). These data are in agreement with the previously reported association constant for the OTB–HSA complex (*K_a_* = 1.80 × 10^6^ L/mol) by Perry et al. [22]. This finding demonstrates that the stability of OTC–HSA is one magnitude higher compared to the OTB–HSA complex (Table 1). Thus, OTB and OTC form significantly less and similarly stable complexes with HSA than ochratoxin A, respectively. Nevertheless, even the binding affinity of OTB toward HSA can be considered high. Based on these observations, both the absence of chlorine substituent (OTB) and the presence of ethyl substituent (OTC) decrease the stability of ochratoxin–HSA complexes. Previous data indicate extremely strong interaction of ochratoxin A with HSA, and the following *K_a_* values have been described: 3.7 × 10^6^ L/mol [35], 5.2 × 10^6^ L/mol [22,36], 5.3 × 10^6^ L/mol [37], 5.6 × 10^6^ L/mol [30], 8.9 × 10^6^ L/mol [38], 2.6 × 10^7^ L/mol [39], and 4.5 × 10^7^ L/mol [40]. The stability of ochratoxin A–albumin complexes decreases in the following order: HSA > bovine > porcine > chicken > rat serum albumin [41,42,43,44].

### 2.2. Spectroscopic Investigation of the Interactions of PAT, DON, and T2 with HSA

First, the HSA binding of PAT, DON, and T2 were evaluated by fluorescence quenching method. Even high concentrations of DON and T2 (15 μM mycotoxin vs. 2 μM albumin) did not modify the fluorescence emission spectrum of HSA (data not shown), which makes questionable their complex formation with albumin. Interestingly, a previous study suggested the strong quenching effect of DON on HSA [24]. Yuqin et al. [24] applied fatty acid-free HSA; however, it is unlikely that fatty acids can cause such a large difference. In that study, the inner filter effect of DON was not corrected, and a second peak appeared at around 450 nm [24], where a band is not typical regarding HSA or DON. It may have resulted from the low purity of the applied protein.

**Figure 6:**
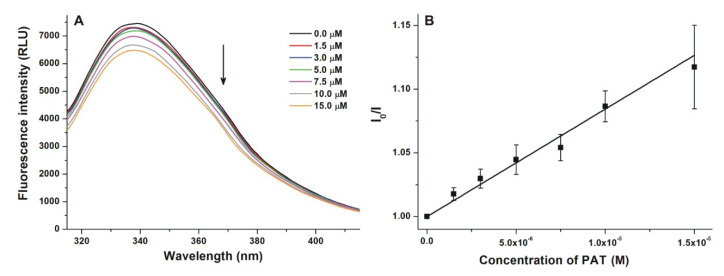
(**A**) Fluorescence emission spectrum of human serum albumin (HSA; 2 μM) in the presence of increasing concentrations (0–15 µM) of patulin (PAT) in phosphate-buffered saline (PBS, pH 7.4). (**B**) Stern–Volmer plot of PAT–HSA interaction (λ_ex_ = 295 nm, λ_em_ = 340 nm; ex slit = 10 nm, em slit = 10 nm; RLU: relative light unit).

PAT slightly decreased the emission signal of HSA in a concentration-dependent fashion (Figure 6A). The inner filter effect of the mycotoxin was corrected (see in Equation (1)), therefore, this observation suggests the interaction of PAT with HSA. The Stern–Volmer plot displayed good linearity (Figure 6B, R^2^ = 0.99), the *K_SV_* value was 8.23 × 10^3^ (±1.12 × 10^3^) L/mol. The data analysis with the Hyperquad2006 software also yielded a relatively low association constant (*K_a_* = 1.38 × 10^4^ ± 0.08 × 10^4^ L/mol). In a previous study, somewhat higher *K_SV_* (1.40 × 10^4^ L/mol) and *K_a_* (4.59 × 10^4^ L/mol) values have been reported [25]. However, Yuqin et al. did not correct the inner filter effect of the mycotoxin, which can cause significant error in the presence of high PAT concentrations (such as 30 μM used in that study) [25]. Another report suggests the weak interaction of PAT with bovine serum albumin [45]. 

The association constant of PAT–HSA complex is three orders of magnitude lower compared to ochratoxin A–HSA (*K_a_* ≈ 3 × 10^7^ L/mol) [21] and approximately one magnitude lower than zearalenone–HSA (*K_a_* = 1.2 × 10^5^ L/mol), citrinin–HSA (*K_a_* = 2.0 × 10^5^ L/mol), and alternariol–HSA (*K_a_* = 4.0 × 10^5^ L/mol) complexes [18,19,20]. Nevertheless, PAT binds to HSA with similar affinity than aflatoxins (the reported *K_a_* values are 1 × 10^4^ to 6 × 10^4^ L/mol) [27,29].

**Figure 7:**
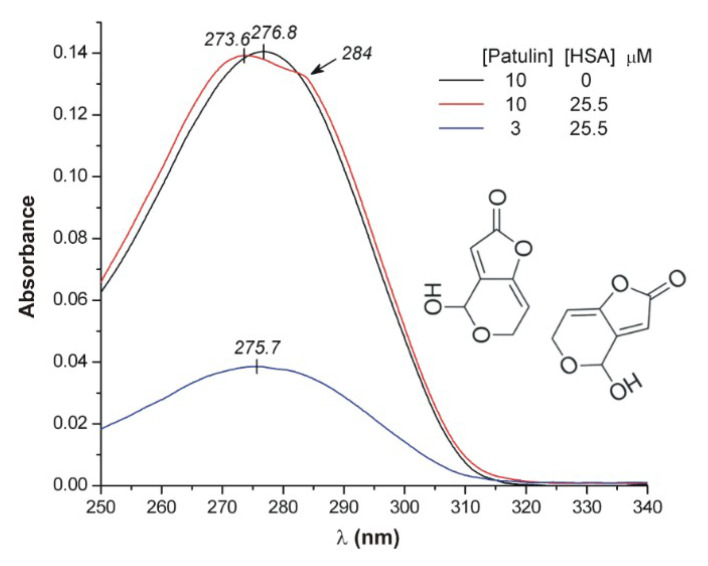
Comparison of the UV absorption spectra of patulin (PAT) recorded in the absence and presence of 25 μM human serum albumin (HSA; pH 7.4, 25 °C, optical path length: 1 cm). A possible relative steric arrangement of two PAT molecules at the HSA binding site is shown. Long axes of the conjugated π-systems are nearly perpendicular to each other, resulting in the exciton splitting of the UV band.

Since PAT, DON, and T2 do not display intrinsic fluorescence, these interactions were also examined employing CD and UV spectroscopy. Binding of a ligand molecule within the chiral protein matrix often induces CD bands which cannot be observed under protein-free conditions [16,46,47]. These so called Cotton effects (CEs) are associated with the π–π* and/or n–π* electronic transitions of the guest compound. The n–π* band of the ester moiety of PAT is below 240 nm, where the strong masking effect of the intrinsic CD as well as UV absorption activity of HSA hampers the detection of induced CEs. Above 250 nm, however, PAT exhibits a π–π* type UV peak centered at 277 nm (Figure 7). In this region, the CD signals of albumin are much weaker, allowing the study of the binding of the mycotoxin. Accordingly, a titration experiment was conducted, increasing the PAT/HSA molar ratio from zero to 3.6, but no induced CD features were observed (data not shown). Contrary to this, some UV spectral changes could be noticed. In relation to the protein-free state of PAT, the bell-shaped, symmetrical absorption band slightly broadens in the presence of HSA and displays two, partially resolved maxima around 274 and 284 nm (Figure 7). Such a kind of band splitting can be rationalized by the exciton theory [48,49,50]. If two ligand molecules are accommodated close to each other at a common protein binding site, excited state interactions may take place between their π–π* transitions resulting in two—a higher- and a lower-energy level—exciton states. Intensity of these exciton components varies depending on the relative steric disposition of the molecules (more exactly, the π–π* transition dipole moments). In our case, their almost equal amplitudes indicate that long axes of the excitonically coupled PAT molecules bound simultaneously at the same site are orthogonal to each other, i.e., I_ (Figure 7). In the very initial phase of the titration, the HSA binding site of PAT is in a large molar abundance relative to the toxin concentration. Therefore, the binding equilibrium is still shifted to the 1:1 PAT–HSA complex, and thus, the UV band shows no splitting pattern (see the blue curve in Figure 7). Upon further increase of toxin concentration, 2:1 PAT-HSA ternary complexes will prevail with the consequent UV band split. Seemingly, it is surprising that there is no induced CD activity despite of the co-binding of two PAT molecules and the exciton interaction between their conjugated π-systems. Ternary complexes of HSA and other proteins often exhibit a characteristic biphasic CD band pair generated by the chiral exciton coupling mechanism [47,51,52,53]. For such result, however, the coupled transition dipole moments must be located in different planes. Taking into consideration that no induced CEs were detected, it is proposed that the π-systems of PAT molecules bound at the HSA site are nearly co-planar.

**Figure 8:**
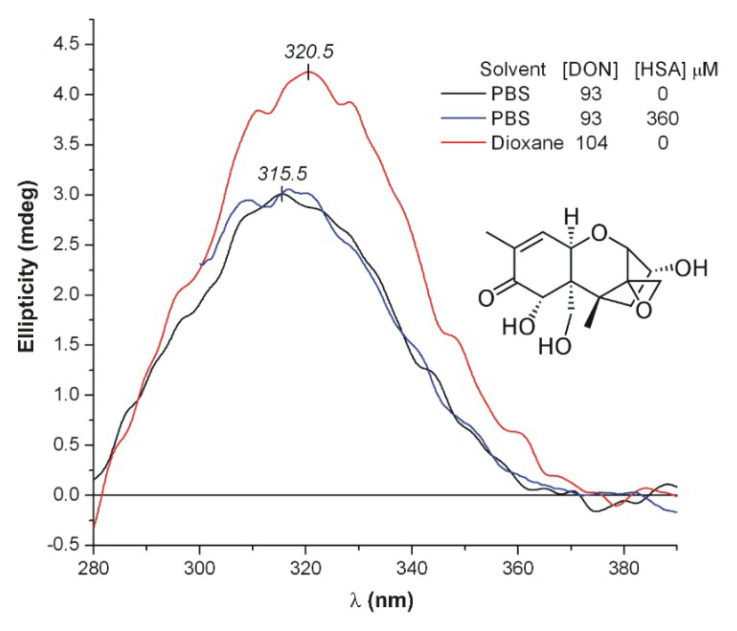
Comparison of the intrinsic n–π* CE of deoxynivalenol (DON) measured in dioxane and in phosphate-buffered saline (PBS, pH7.4) with human serum albumin (HSA) and under protein-free condition (25 °C, optical path length: 1 cm).

DON contains a conjugated carbonyl chromophore, the n–π* transition of which is CD active, showing a broad, positive CE above 280 nm (Figure 8). The λ_max_ value of the n–π* CD band of saturated and unsaturated ketones is sensitive to the polarity of the environment [54,55,56]. In polar aqueous medium, the n–π* CD maximum of DON is at 315.5 nm, whereas it shifts to 320.5 nm in the apolar, aprotic solvent dioxane (Figure 8). Similarly, inclusion of DON into a less polar, hydrophobic HSA binding cavity should also shift its CD extremum to longer wavelengths. This kind of red shift can be used as a diagnostic tool for protein binding of chiral compounds owing a carbonyl moiety [57]. However, the spectral position of the CD bands obtained in protein-free buffer solution and at high molar excess of HSA are practically the same (Figure 8). Thus, it seems that DON does not enter into any hydrophobic cleft of the protein.

Due to the presence of several chiral centers, T2 also exhibits intrinsic CD signals which are associated with the π–π* and n–π* electronic transitions of the ethene and ester chromophores. These CD bands can be measured below 240 nm, and thus, do not interfere with the near-UV CD motif of HSA, which originates from the chiral perturbation of the aromatic side chains and might be sensitive to the binding of ligand molecules [57]. To test this option, the near-UV CD spectrum of 24 µM HSA was monitored between 245–340 nm during the increase of toxin concentration from zero to 137 µM. However, unambiguous and relevant spectral modifications could not be registered (data not shown). In addition, to study whether the secondary structure of the protein is affected by the presence of T2, the far-UV CD region was also scanned. Apart from the additive intrinsic CD contribution of the toxin, there was no significant difference between the CD curves measured for free HSA and HSA+T2 mixture (1.5 µM HSA and 535 µM toxin; data not shown).

### 2.3. Effects of Mycotoxins on the Albumin Binding of Site I and II Markers Based on Ultrafiltration

**Figure 9:**
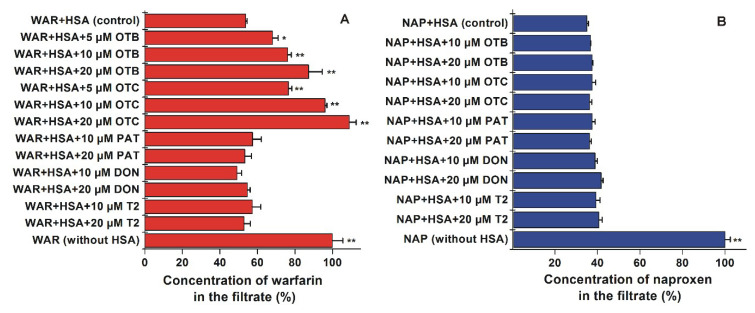
Concentration of (±)-warfarin (WAR; (**A**)) and (±)-naproxen (NAP; (**B**)) in the filtrate. Samples contained warfarin and HSA (1.0 and 5.0 μM, respectively) or naproxen and HSA (1.0 and 1.7 μM, respectively) with and without mycotoxins (OTB, ochratoxin B; OTC, ochratoxin C; PAT, patulin; DON, deoxynivalenol; T2, T-2 toxin; human serum albumin, HSA; ** *p* < 0.01, * *p* < 0.05).

Ultrafiltration experiments were carried out using (±)-warfarin (site I) and (±)-naproxen (site II) as site markers [14]. Albumin-bound molecules are entrapped in the retentate due to the large size of the protein; however, free (unbound) ligands pass through and consequently appear in the filtrate. Therefore, the increased concentrations of site markers in the filtrate is resulted from their displacement from HSA [20,58]. In a concentration-dependent fashion, both OTB and OTC significantly increased the warfarin content in the filtrate, suggesting its displacement from the site I (Figure 9A). In line with the case of ochratoxin A [30], the high-affinity binding site of OTB and OTC is located in subdomain IIA. The higher displacing effect of OTC compared to OTB is in accord with the higher stability of OTC-HSA (see in Section 2.1). However, PAT, DON, and T2 did not alter the concentration of warfarin in the filtrate, even at 20-fold concentrations vs. the site marker. Furthermore, none of the tested mycotoxins affected the interaction of the site II marker naproxen with HSA (Figure 9B). Previous results validate this model since under the same experimental conditions, flavonoids chrysin, chrysin-7-sulfate, and 7,8-dihydroxyflavone (*K_a_* values of these albumin–ligand complexes are 2.5 × 10^5^ to 7.6 × 10^5^ L/mol) caused the statistically significant displacement of naproxen [58,59]. Thus, ultrafiltration studies also confirm the strong interactions of OTB and OTC with the site I region of HSA. Furthermore, these observations demonstrate the poor displacing ability of PAT, DON, and T2 vs. site I and II ligands.

### 2.4. Effect of Albumin on the Acute Cellular Toxicity of Mycotoxins

**Figure 10:**
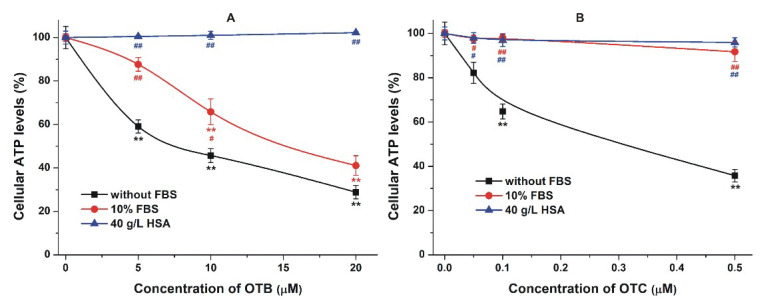
Effects of ochratoxin B (OTB; (**A**)) and ochratoxin C (OTC; (**B**)) on adenosine triphosphate (ATP) levels/well (% of control). HepG2 cells were incubated for 48 h in the absence and presence of 10% fetal bovine serum (FBS) or 40 g/L human serum albumin (HSA; statistical significance compared to the corresponding control: ** *p* < 0.01; compared to the effect without albumin: ^#^ *p* < 0.05, ^##^ *p* < 0.01).

**Figure 11:**
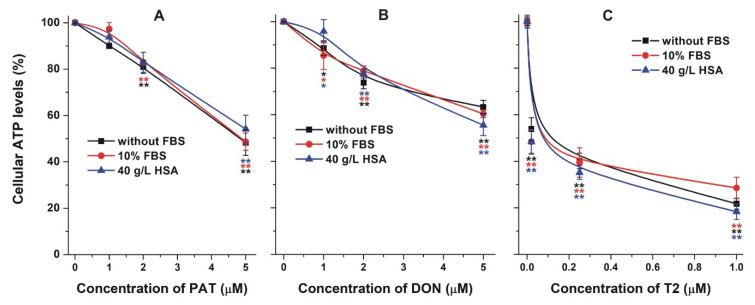
Effects of patulin (PAT; (**A**)), deoxynivalenol (DON; (**B**)), and T-2 toxin (T2; (**C**)) on the cellular adenosine triphosphate (ATP) levels/well (% of control). HepG2 cells were incubated for 48 h in the absence and presence of 10% fetal bovine serum (FBS) or 40 g/L human serum albumin (HSA; statistical significance compared to the corresponding control: * *p* < 0.05, ** *p* < 0.01).

As has been demonstrated previously, albumin can strongly alleviate the in vitro toxic effects of certain mycotoxins (e.g., citrinin and ochratoxin A) [18,34,60], via the limitation of their tissue uptake [35]. To test the toxicological importance of mycotoxin–albumin interactions, the cytotoxicity of OTB, OTC, PAT, DON, and T2 were examined without and with 10% fetal bovine serum (FBS) or 40 g/L HSA on HepG2 cells. The toxic effects of mycotoxins were evaluated employing ATP-based cell viability assay. In the absence of albumin, mycotoxins strongly decreased ATP levels/well (Figure 10 and Figure 11). 

In agreement with previous data, OTB exerted considerably lower toxicity than OTC [4,61]. In the presence of albumin, the toxic effects of OTB and OTC declined (Figure 10). FBS (10%, which means approximately 3.5 g/L bovine serum albumin in the cell culture medium) significantly reduced and abolished the OTB- and OTC-induced viability loss, respectively. Moreover, in the presence of 40 g/L HSA, the same concentrations of OTB and OTC did not affect cellular ATP levels. These results suggest that the formation of highly stable OTB-HSA and OTC-HSA complexes diminishes the cellular uptake of ochratoxins, indicating the significant toxicokinetic importance of these interactions.

The cytotoxic effect of T2 was considerably higher compared to PAT or DON, which is in good agreement with the previously reported data [62]. The presence of FBS or HSA did not affect the toxic effects of PAT, DON, and T2 (Figure 11). In the lack of formation of high-affinity mycotoxin–albumin complexes, albumin was unable to reduce the cellular uptake of these mycotoxins.

## 3. Conclusions

Based on fluorescence studies, OTB and OTC form highly stable complexes with HSA. Fluorescence quenching and CD experiments suggest the low-affinity interaction of PAT with HSA, while DON and T2 likely do not interact with the protein or form poorly stable complexes. In ultrafiltration experiments, OTB and OTC significantly displaced the site I marker warfarin but other mycotoxins tested did not affect the albumin binding either of warfarin or naproxen. Cytotoxic effects of OTB and OTC were alleviated or even abolished in the presence of albumin, suggesting the high toxicokinetic importance of OTB–HSA and OTC–HSA complex formations. Since albumin binding of mycotoxins can strongly affect their tissue distribution and elimination half-life, these results may help deepen the understanding of the toxicokinetics of mycotoxins. Cell experiments help to explore the toxicological importance of mycotoxin–albumin interactions, because several other factors (e.g., diffusibility of the compound and the involvement of active transport mechanisms) can also influence the cellular uptake of mycotoxins from the circulation. Due to the complex toxicokinetics of these compounds, it is reasonable to perform animal studies in the future for the better characterization of mycotoxin–albumin interactions. For example, the displacement of highly albumin-bound mycotoxins (e.g., ochratoxins) from the protein may strongly modify their toxicokinetics and toxicity.

## 4. Materials and Methods

### 4.1. Reagents

Patulin (PAT), deoxynivalenol (DON), T-2 toxin (T2), human serum albumin (HSA; product code: A1653; containing fatty acids), racemic warfarin, racemic naproxen, and Dulbecco’s Modified Eagle Medium (DMEM) were purchased from Sigma-Aldrich (St. Louis, MO, USA). The bioluminescent ATP Assay Kit CLSII (from Roche, Basel, Switzerland) and fetal bovine serum (FBS; from Pan-Biotech GmbH, Aidenbach, Germany) were used as received. Ochratoxin B (OTB) and ochratoxin C (OTC) were purchased from Cfm Oskar Tropitzsch GmbH (Marktredwitz, Germany) and from BioMarker Ltd. (Gödöllő, Hungary), respectively. Spectroscopic and ultrafiltration studies were carried out in phosphate-buffered saline (PBS, pH 7.4).

### 4.2. Fluorescence Spectroscopic Measurements

Fluorescence and UV spectra were recorded at 25 °C in the presence of air, employing a Hitachi F-4500 spectrofluorometer (Tokyo, Japan) and a Specord Plus 210 spectrophotometer (Analytik Jena; Jena, Germany), respectively. The inner filter effects of mycotoxins were corrected using the following equation [63]:

(1) $$I_{cor}=I_{obs}\times e^{\left(A_{ex}+A_{em}\right)/2}$$

where *I_cor_* is the corrected fluorescence emission intensity, *I_obs_* is the observed emission signal, while *A_ex_* and *A_em_* denote the absorbance of mycotoxins at the excitation and emission wavelengths used, respectively.

In fluorescence quenching studies, emission spectra of HSA (2 μM) were recorded in the absence and presence of mycotoxins (final concentrations of PAT, DON and T2: 0.0, 1.5, 3.0, 5.0, 7.5, 10.0, and 15.0 µM; final concentrations of OTB and OTC: 0, 0.1, 0.25, 0.5, 0.75, 1.0, 1.25, 1.5, and 2.0 µM) using 295 nm excitation wavelength. Under the applied conditions, solvents applied (dimethyl sulfoxide or ethanol) did not affect the fluorescence emission signal of HSA at 340 nm. Association constants (*K_a_*) were determined using the Hyperquad2006 software (Version 3.1.60, Protonic Software, Leeds, UK) by non-linear fitting, as described [23,34,64]. Data were also evaluated based on the Stern–Volmer equation [63]:

(2) $$\frac{I_{0}}{I}=1+K_{SV}\times \left[Q\right]$$

where *I*_0_ is the fluorescence emission intensity of HSA without mycotoxins, *I* is the emission signal of HSA in the presence of mycotoxins (λ_ex_ = 295 nm, λ_em_ = 340 nm), *K_SV_* is the Stern–Volmer quenching constant, and [*Q*] is the molar concentration of the quencher.

Fluorescence emission spectra of ochratoxins (1 µM) were also examined in the presence of increasing concentrations of HSA (0–7.5 µM for OTB and 0–3 µM for OTC). The emission spectra were recorded using 295 nm excitation wavelength, because of the energy transfer between HSA and ochratoxins [23]. Furthermore, the fluorescence excitation wavelength maxima of OTB (365 nm) and OTC (380 nm) were also applied, to examine the changes in the fluorescence emission signals of ochratoxins resulting from their interaction with HSA. Association constants of OTB–HSA and OTC–HSA complexes were determined by the Hyperquad2006 software, as have been reported previously [23]. Fluorescence anisotropy measurements were performed with the same samples, at the wavelength maxima of OTB (λ_ex_ = 365 nm, λ_em_ = 430 nm) and OTC (λ_ex_ = 380 nm, λ_em_ = 444 nm). Anisotropy (*r*) values were calculated using the following equation [23]:

(3) $$r=\frac{\left(I_{VV}-G\times I_{VH}\right)}{\left(I_{VV}+2\times G\times I_{VH}\right)}$$

where *G* denotes the instrumental correction factor, while *I_VV_* and *I_VH_* show the fluorescence emission intensities determined in vertical position of polarizer at the pre-sample site and at vertical and horizontal positions of the post-sample polarizer, respectively. Association constants were calculated from anisotropy values as described previously [23].

### 4.3. Circular Dichroism (CD) and Absorption Spectroscopic Measurements

CD and UV absorption spectra were acquired at 25 ± 0.2 °C on a JASCO J-715 spectropolarimeter (Tokyo, Japan) equipped with a Peltier thermostat. All spectra were monitored in continuous scanning mode at a rate of 50 nm/min, with a step size of 0.1 nm, response time of 2 s, three accumulations and 1 nm bandwidth, using 1 or 0.1 cm path-length quartz cuvette (Hellma GmbH & Co., Plainview, NY, USA). Absorption spectra were obtained by conversion of the high tension (HT) voltage applied to the photomultiplier tube into absorbance units. CD and absorption curves of mycotoxin–HSA mixtures were corrected by spectral contribution of blank HSA solutions. Albumin samples were dissolved in PBS (pH 7.4). The concentration of HSA (MW: 66,500) was determined using its weighted amount. Stock solutions of mycotoxins were prepared as follows: DON, 8.4 mM in deionized water; T2, 5.4 mM in ethanol; PAT, 2.6 mM in deionized water. To test the albumin binding of PAT and T2, 1.8 mL HSA samples were prepared at 25 and 24 µM concentrations, respectively. After recording the CD and UV absorption spectra of free HSA, µL aliquots of the stock solutions of the mycotoxins were pipetted consecutively into the protein sample. CD and absorption curves were scanned after addition of each aliquot. In the case of DON, the mycotoxin was mixed into 0.36 mM HSA solution.

### 4.4. Ultrafiltration

To investigate the effects of mycotoxins on the albumin binding of Sudlow’s site I and II ligands, ultrafiltration experiments were performed as described earlier [20,58]. Briefly, samples contained warfarin–HSA (1.0 and 5.0 μM, respectively) or naproxen–HSA (1.0 and 1.5 μM, respectively) mixtures in the absence and presence of mycotoxins (5, 10, or 20 µM) in PBS (pH 7.4). Ultrafiltration (10 min, 7500 g, and 25 °C) was performed using Pall Microsep™ Advance Centrifugal Devices (30 kDa molecular weight cut-off value; VWR, Budapest, Hungary), then the concentrations of warfarin and naproxen in the filtrate were quantified by HPLC. 

The HPLC system was built up from a Waters 510 HPLC pump (Waters, Milford, MA, USA), a Rheodyne 7125 injector (Rheodyne, Berkeley, CA, USA) linked to a 20 µL sample loop, a Waters 486 UV detector, and a Jasco FP-920 fluorescent detector (Jasco, Tokyo, Japan). Peak areas were evaluated employing Waters Millennium Chromatography Manager software (Version 3.2, Waters Corporation, Milford, MA, USA). Warfarin and naproxen were quantified as described previously [20]. The limit of detection (LOD) and the limit of quantification (LOQ) were the lowest concentrations when the signal-to-noise ratios 3 and 10 were observed, respectively. The LOD values were 0.05 μM for both warfarin and naproxen. The LOQ values were 0.1 and 0.2 μM for warfarin and naproxen, respectively. Both methods showed good linearity (warfarin: R^2^ = 0.998; naproxen: R^2^ = 0.999) in the concentration range of 0.05–2.00 μM. Reproducibility was established by intraday and interday precision. The intraday coefficients of variation values were 2.1% for warfarin and 1.0% for naproxen (n = 7). The interday coefficient of variation values were 6.4% and 5.4% for warfarin and naproxen, respectively (n = 5).

### 4.5. Cell Culturing and Viability Assay

Cell experiments were performed on HepG2 (human hepatocellular carcinoma; ATCC: HB-8065) adherent cell line. The cells were cultured in DMEM with 10% FBS, 100 U/mL penicillin, and 100 µg/mL streptomycin (5% CO_2_, 37 °C). Cells (10^4^/well in 96-well plates) were treated for 48 h with OTB (5.0, 10.0 and 20.0 µM), OTC (0.05, 0.1, and 0.5 µM), PAT (1.0, 2.0, and 5.0 µM), DON (1.0, 2.0, and 5.0 µM), and T2 (0.02, 0.25, and 1.0 µM) in the absence and presence of 10% FBS or 40 g/L HSA. ATP levels were quantified applying the previously described method without modifications [65].

### 4.6. Statistics

Data demonstrate the mean and standard error of the mean (SEM) values. Assays were performed at least in triplicate. For statistical evaluation, the one-way ANOVA (IBM SPSS Statistics, v. 21, New York, NY, USA; *p* < 0.05 and *p* < 0.01) was applied, employing Tukey’s post hoc test.

## Figures and Tables

**Table 1:** Stern–Volmer quenching constants (*K_SV_*; unit: L/mol) and association constants (*K_a_*; unit: L/mol) of ochratoxin–HSA complexes based on fluorescence spectroscopic studies.

Complex	*K_SV_* (±SEM) [×10^6^ L/mol] (Stern–Volmer Plot, Figure 2)	*K_a_* (±SEM) [×10^6^ L/mol] (Hyperquad, Figure 2)	*K_a_* (±SEM) [×10^6^ L/mol] (Hyperquad, Figure 3)	*K_a_* (±SEM) [×10^6^ L/mol] (Hyperquad, Figure 4)	*K_a_* (±SEM) [×10^6^ L/mol] (Anisotropy, Figure 5)
OTB–HSA	0.57 ± 0.05	0.83 ± 0.08	1.38 ± 0.21	1.02± 0.13	0.92 ± 0.04
OTC–HSA	0.46 ± 0.06	0.77 ± 0.11	16.02 ± 4.7	9.36 ± 2.00	6.82 ± 0.24

*OTB*, ochratoxin B; *OTC*, ochratoxin C; *HSA*, human serum albumin; *SEM*, standard error of the mean.
